# 2025: A Year of Great Success for *Muscles*

**DOI:** 10.3390/muscles4040046

**Published:** 2025-10-17

**Authors:** Guglielmo Duranti

**Affiliations:** Laboratory of Biochemistry and Molecular Biology, Department of Movement, Human and Health Sciences, Università degli Studi di Roma “Foro Italico”, Piazza Lauro de Bosis, 600135 Rome, Italy; guglielmo.duranti@uniroma4.it; Tel.: +39-0636733479

MDPI’s *Muscles* is celebrating a highly positive 2025.

Since its founding in 2022 [1,2], this journal has attracted growing interest from the international community. This has aligned with the increased focus on muscle and its pathophysiology that has developed in recent years [3].

Skeletal muscle constitutes approximately 40% of total body weight in adults, and its health is essential for overall well-being. Muscle is responsible for movement, maintaining posture, supporting bones, regulating energy metabolism, and protecting the body—all fundamental functions. The health of this tissue is therefore crucial for maintaining individual health. Consequently, great attention on this topic has emerged within international scholarship (Figure 1) [3].

In this context, *Muscles* has been significant, responding to scholarly interest and providing an effective platform to present recent and innovative research in this field. Since its foundation in 2022, the numbers of submissions and published articles have grown steadily (Figure 2).

In particular, during 2025, the first historic milestone was reached for *Muscles*: the publication of its hundredth article [4]. This is highly significant for a journal that was only founded in 2022.

Among the various published articles, some have achieved significant relevance in terms of citations, reading by individuals and researchers, and numbers of downloads [5].

In terms of citation counts, as of 1 October 2025, the most cited articles were “Myokines in Appetite Control and Energy Balance” by Grannel et al. and “The Role of Mitochondria in Mediation of Skeletal Muscle Repair” by Alway et al., both with 23 citations [6,7] (Figure 3).

In terms of article access numbers, the two most viewed articles were “The Effects of Multiple Acute Turkesterone Doses on Indirect Measures of Hypertrophy and Metabolic Measures: A Preliminary Investigation” by Harris et al., accessed 50,336 times, and “Effects of Detraining on Muscle Strength and Hypertrophy Induced by Resistance Training: A Systematic Review” by Encarnação et al., accessed 29,856 times [8,9] (Figure 4).

Additionally, the most downloaded articles were Santos and Haluch’s “Downregulation of Androgen Receptors upon Anabolic-Androgenic Steroids: A Cause or a Flawed Hypothesis of the Muscle-Building Plateau?”, with 3207 downloads, and Moreira et al.’s “Analysis of Muscle Strength and Electromyographic Activity during Different Deadlift Positions” with 1921 downloads [10,11] (Figure 5).

Finally, another major milestone was reached by *Muscles* in 2025: indexing in major research reference systems, including PubMed and Scopus [12]. This of great significance, as publications being listed in these systems and thus receiving greater visibility will be a notable incentive for future submissions, which will continue to enhance the journal’s visibility and reputation.

Furthermore, the journal’s initial impact factor is pending, which will hopefully provide even more impetus for submissions [12].

However, much remains to be carried out in terms of promotion to ensure the long-term stability of *Muscles*, even though it currently appears projected towards a bright future.

## Figures and Tables

**Figure 1:**
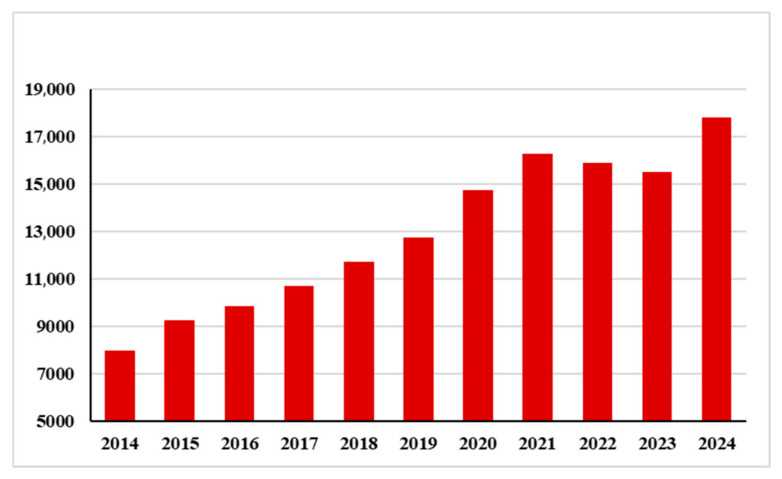
Numbers of publications indexed in PubMed from 2014 to 2024 applying the keyword “muscle” and in combination with “health”.

**Figure 2:**
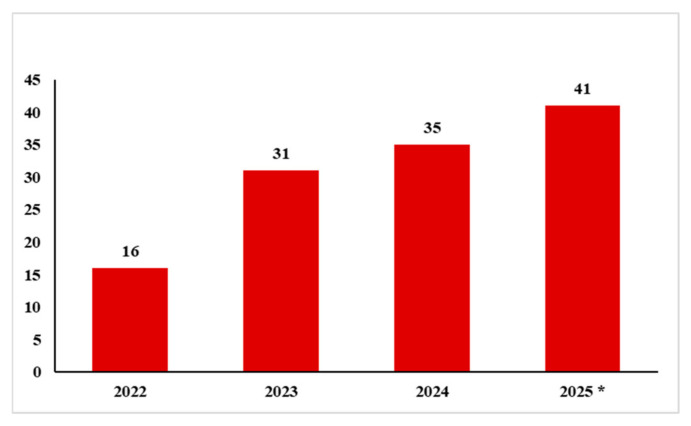
Numbers of publications in *Muscles* from 2022 to 2025. *: number verified as of October 1, 2025.

**Figure 3:**
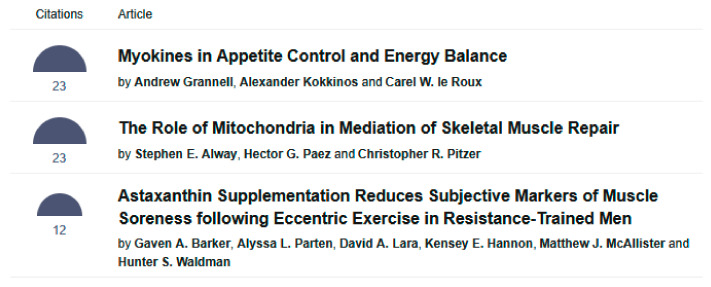
The three most cited articles in *Muscles* as of 1 October 2025 (https://www.mdpi.com/journal/muscles/most_cited, accessed on 1 October 2025).

**Figure 4:**
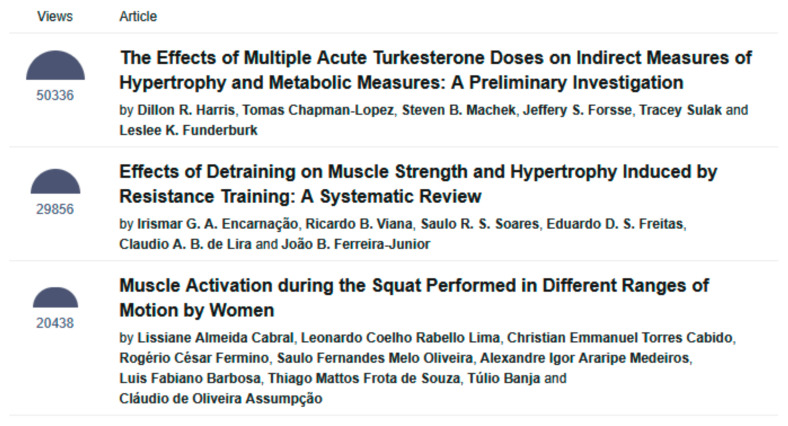
The three most viewed articles in *Muscles* as of 1 October 2025 (https://www.mdpi.com/journal/muscles/most_cited, accessed on 1 October 2025).

**Figure 5:**
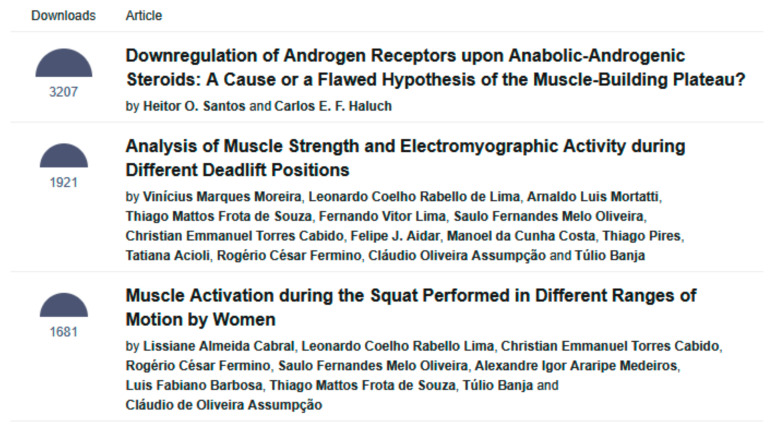
The three most downloaded articles in *Muscles* as of 1 October 2025 (https://www.mdpi.com/journal/muscles/most_cited, accessed on 1 October 2025).
